# Supplementary material for: Real-Time and Ultrasensitive Prostate-Specific Antigen Sensing Using Love-Mode Surface Acoustic Wave Immunosensor Based on MoS2@Cu2O-Au Nanocomposites
Source: Sensors (Basel). 2024 Nov 29;24(23):7636. doi: 10.3390/s24237636 (PMC11644860; doi:10.3390/s24237636)
Supplement: Supplementary file 1 [file sensors-24-07636-s001.zip › sensors-3297660-supplementary.pdf]

Supplementary Information

Real-Time and Ultrasensitive Prostate-Specific Antigen Sensing Using Love-Mode Surface Acoustic Wave Immunosensor Based on MoS_2@Cu_2O-Au Nanocomposites

Yan Yu [1], Haiyu Xie [1], Tao Zhou [1], Haonan Zhang [1], Chenze Lu [2], Ran Tao [1,*], Zhaozhao Tang [3,*] and Jingting Luo [1]

[1] Shenzhen Key Laboratory of Advanced Thin Films and Applications, GuangDong Engineering Technology Research Centre of Breath Test, College of Physics and Optoelectronic Engineering, Shenzhen University, Shenzhen 518060, China; 2200451022@email.szu.edu.cn (Y.Y.); 1810342119@email.szu.edu.cn (H.X.); 2200453042@email.szu.edu.cn (T.Z.); 2300451008@email.szu.edu.cn (H.Z.); luojt@szu.edu.cn (J.L.)

[2] Key Laboratory of Specialty Agri-Products Quality and Hazard Controlling Technology of Zhejiang Province, College of Life Sciences, China Jiliang University, Hangzhou 310018, China; chenzelu@cjlu.edu.cn

[3] Water Science and Environmental Engineering Research Centre, College of Chemistry and Environmental Engineering, Shenzhen University, Shenzhen 518060, China

* Correspondence: ran.tao@szu.edu.cn (R.T.); zhaozhao.tang@foxmail.com (Z.T.)

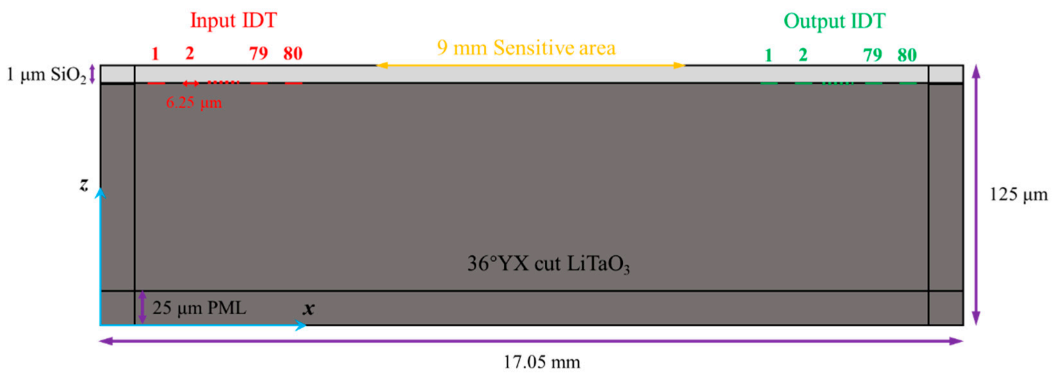

Figure S1. 2D computational domain for L-SAW immunosensor.

Table S1. Parameters of the L-SAW sensor chip

IDT material	Au
Finger width (μm)	6.25
Finger period (μm)	25
Finger pairs	40
IDT thickness (nm)	100
Sound velocity (m/s)	4212
Sound aperture (μm)	3.5
Length of delay line area (mm)	15
Centre frequency (MHz)	175.5

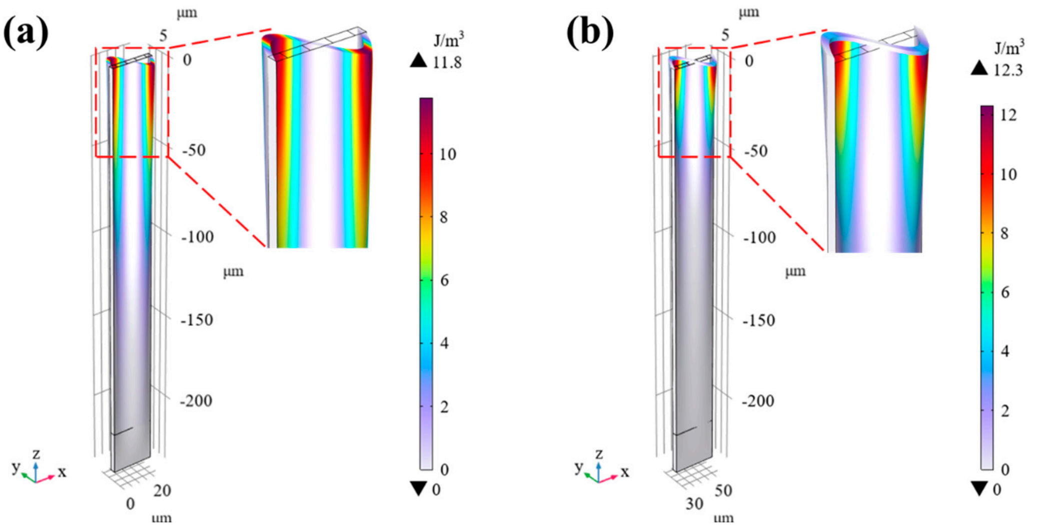

Figure S2. Kinetic energy density distribution in simulations (a) without waveguide layer; (b) with SiO_2 waveguide layer.

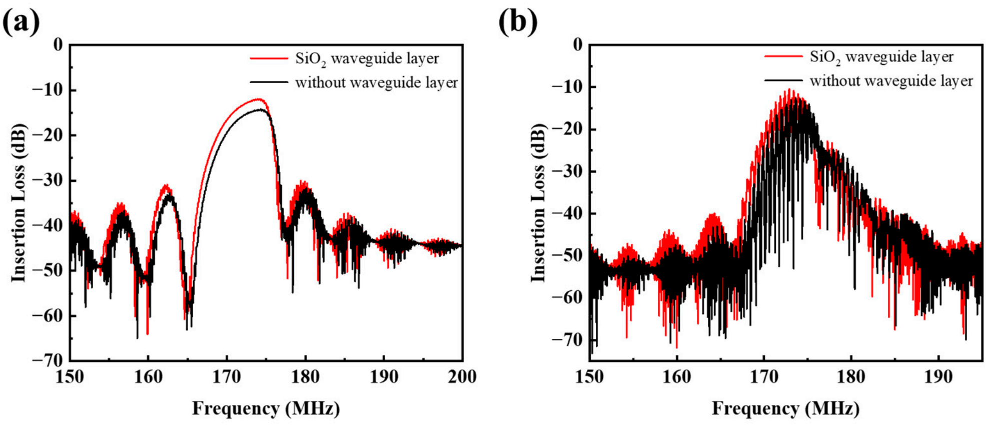

Figure S3. (a) Comparison of S_{21} signals with and without SiO_2 waveguide layer in simulation; (b) comparison of S_{21} signals from the fabricated immunosensors with and without SiO_2 waveguide layer.

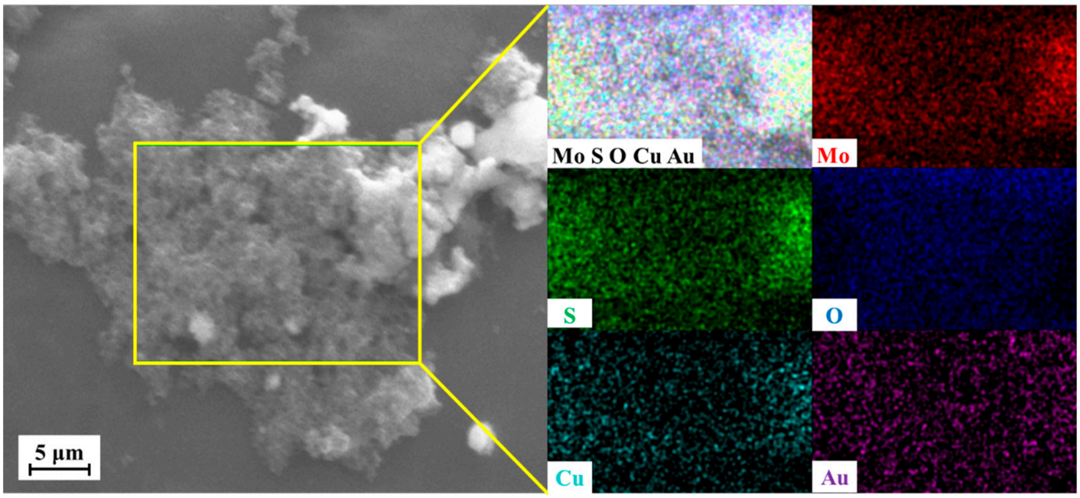

Figure S4. The EDS map scanning analysis of $MoS_2@Cu_2O$-Au.

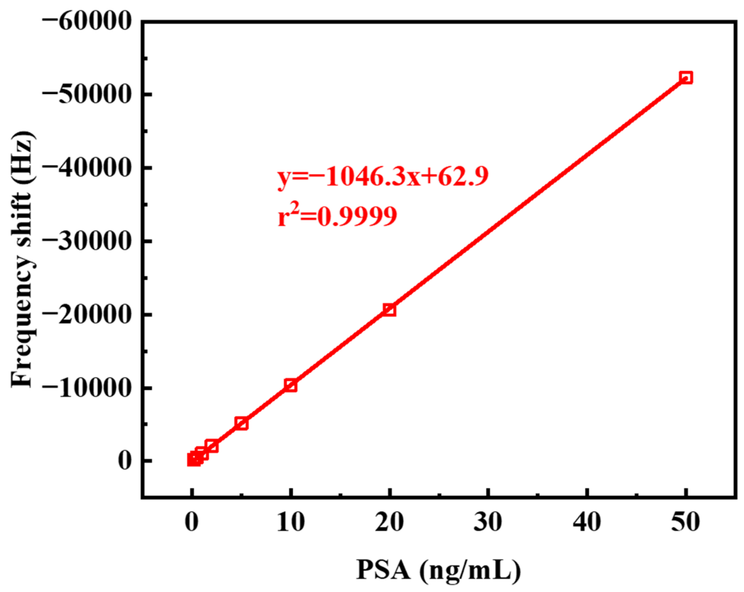

Figure S5. Simulated results of frequency shift with the incremental PSA concentration.
